# The Struggle to Rise

**Published:** 1919-03-08

**Authors:** 


### THE STRUGGLE TO RISE.

Nobody will dispute the statement that wherever one turns one's attention is arrested by a lively endeavour of nearly every social class for more of its own way, more of its own pay, and less of its own routine work. None of them wants (to take perhaps rather a low view of human motives) to be left behind just now, to miss the chances of general betterment that the great upheaval, to be felt for many a long day yet, has made possible; in plenty of cases, indeed, undeniably desirable too in the interests of all. This phenomenon, the ground swell of the war, must be viewed broadly and without fear or favour. It would be absurd, and—were not that the national vice—hypocritical past bearing, to denounce working men for "unrest," when almost all the members of our own well-loved medical household are in the same condition of keen agitation, of Brownian movement—when nurses are combining against under-payment and under-staffing; when a general practitioner's loud call for a trades' union and the strike weapon is enthusiastically acclaimed by his fellows, despising the staider counsel of their seniors; when (to take the latest instance) tuberculosis officers demand autonomy and considerably increased salaries. A great deal has been successfully hushed up this last four years, but the present movement is too widespread now even for that handy measure; while to try to scold it down is preposterous. Perhaps the first thing the responsible person might do is not to let everybody—we say everybody—take up unhindered the position of the injured party. It might be pointed out, truly if tritely, that most classes have their little shortcomings. That, for instance, the uniform enforced upon nurses is an absurd one, and that the fetish of a system which robs invalids of their night's rest will have to undergo disenchantment pretty soon; that, again, the long-standing commercialisation of general medical practice would arouse formidable lay indignation were it competently exposed by some one ready to stand trials for libel; also that many municipal medical officers have been appointed for reasons that would not bear close investigation a minute. Still, whatever the tactics employed, the demand for more expenditure cannot be checked to any great extent; and the anxious question which the most elementary common sense is continually suggesting is that very, very old, very, very familiar one which every individual who wants to get through life successfully must put to himself a good many times—Where is the money to come from?

All very well to rejoin "It came quickly enough, from somewhere, for the war." But then it went quickly enough too. What we have now, to speak plain truth, is a country with lots of ships and cargoes sunk, with some of the money lent to numerous allies as good as lost, with a thumping war debt, with an army to maintain (as at present proposed) ten times its pre-war size and at thrice the pre-war pay, with pensions to disburse for the next fifty years, with a notable increase in sickness, with one great trade rival temporarily crushed but with others much advantaged. It is on top of all this that there comes the unanimous demand for looser purse-strings that we have outlined. Happily a good deal of such desiderated expenditure—and what everybody wants usually gets done, the politician but follows public opinion—may prove remunerative after all. As regards our own medical department of human activity, it is comforting to think that a healthier nation must almost necessarily be a more prosperous one. Nevertheless, and for all that, a fool can see that the year 1914 began an era of most thorough-going redistribution of wealth, and that not ending with the "army contractor's only daughter" of the song. For if some must be paid more, then in the long run others have to be paid less: there can be no offence in stating broadly such an obvious necessity. Our concern here is only with the remuneration of the professions, more particularly with that of the learned professions, of which medicine is not now reckoned, as it used to be, the least important. Well, medicine has indeed amply kept up its reputation for progressiveness and beneficence. There does not seem any immediate likelihood of reverting to pre-war expenditure on military and naval affairs, whatever the future may bring forth; or on the higher branches of engineering, with which they are so closely connected. Behind engineering stands applied science, and behind this again the supporter of very much in the life of a modern nation, pure science; these are starved enough, in this country at all events, as it is. Everybody admits that; the endowment of research is a plank in every political platform, from, say, Lord Birkenhead's to Trotsky's. Right, Left, and Centre are all committed to it. So if retrenchment has, as one must suppose it has, to be undertaken at the expense of some profession, it would seem as if that profession cannot be any of those we have yet named. Now there remain, of course, two, one of which, that we propose to leave undesignated, has indeed given proof of an endeavour after economy by seeking to unite its members more closely by their mutual self-sacrifice, a movement which is indeed also amongst the signs of the times. The orthodox process of exclusion is now complete; and in fine this question might be asked at a time of unprecedented financial strain and equally unprecedented medical need: Might not the founding of a Ministry of Health be made to coincide with some reform of the abuses of the law?

